# Assessment of Aleutian mink disease virus (AMDV) prevalence in feral American mink in Iceland. Case study of a pending epizootiological concern in Europe

**DOI:** 10.7717/peerj.12060

**Published:** 2021-09-17

**Authors:** Remigiusz Panicz, Piotr Eljasik, Jakub Skorupski, Przemysław Śmietana, Róbert A. Stefánsson, Menja von Schmalensee, Magdalena Szenejko

**Affiliations:** 1 Department of Meat Technology, Faculty of Food Sciences and Fisheries, West Pomeranian University of Technology Szczecin, Szczecin, Poland; 2 Institute of Marine and Environmental Sciences, Molecular Biology and Biotechnology Centre, University of Szczecin, Szczecin, Poland; 3 West Iceland Nature Research Centre, Stykkisholmur, Iceland

**Keywords:** American mink, Feral population, Free-ranging population, Fulton’s condition factor, Spleen, Virus prevalence and spread

## Abstract

**Background:**

Recurring escapes or deliberate releases and subsequent infiltration or establishment of feral populations by individuals from fur farms have been commonly noted since the beginning of fur industry expansion. Once animals have invaded ecosystems adjacent to source farms escapees can change the demography of the feral populations through hybridization, outbreeding depression, competition and spreading of various pathogens which can decimate wild populations. In our study, we aimed to assess spread of Aleutian mink disease virus (AMDV) in the feral population of American mink (*Neovison vison*) in Iceland. The additional objective was to elucidate whether basic morpho-anatomical parameters (*i.e.*, Fulton’s condition factor or spleen to body weight ratio) might be used as a preliminary indicator of AMDV infection.

**Methods:**

American mink (*n* = 164) were captured by professional hunters in 8 regions of Iceland. The detection of AMDV in the spleen of male and female individuals was based on PCR amplification of an NS1 gene fragment.

**Results:**

We confirmed AMDV presence in 23.8% (*n* = 39) of collected samples with no significant difference in infection rate between males and females. Additionally, we revealed that the prevalence of virus in the feral population was higher closer to fur farms. However, the countrywide prevalence and direction of AMDV distribution needs to be further investigated. Comparison of condition indices in non-infected and infected animals showed significant deterioration of body and spleen parameters in the latter group. Therefore, the application of basic measurements of the American mink may be used to evaluate the health status of individuals in terms of pathogen infection.

**Conclusions:**

The study shed a new light on prevalence and distribution of AMDV in the feral population of American mink in Iceland and the results might be successfully applied to develop models to infer dynamics of various pathogens, even those latently transmitted by disease-free animals.

## Introduction

The American mink (*Neovison vison*) is the main species farmed by the fur industry in Iceland since farms were first established in 1931 (Hersteinsson, 1992; Hersteinsson, 1999; Bonesi & Palazon, 2007). In the following years, excluding the period 1951–1969 when fur-farming was banned by law, events of mink escapes have been recorded (Skírnisson, Gunnarsson & Hjartardóttir, 1990). A countrywide feral population had been established by 1975 (Stefansson, Schmalensee & Skorupski, 2016). Escape events have been confirmed also in *e.g.*, Norway (Bevanger & Henriksen, 1995), Denmark (Pertoldi et al., 2013), Scotland (Macdonald et al., 2015) and France (Fournier-Chambrillon et al., 2004) where American mink, after accidental escapes or deliberate releases, quickly established viable, feral and invasive populations. In the early years of the mink farming industry in Iceland, mink escapes were observed and probably quite common, but the frequency of such events in recent times should be evaluated in the further studies (Stefansson, Schmalensee & Skorupski, 2016). The success of animals in the wild, evidenced by rapid increase of feral populations, has been attributed to the species’ high reproduction capability, opportunistic foraging and the frequent lack of natural enemies and competitors in invaded areas (Bevanger & Henriksen, 1995; Maran et al., 2016). Spread of non-indigenous American mink is associated with a number of serious ecological threats, such as intensive foraging (*e.g.*, small mammals, like *Arvicola amphibious*, ground-nesting birds, fish), competition and aggression (*e.g.*, *Mustela lutreola*) against indigenous fauna (Heggenes & Borgstrom, 1988; Macdonald & Strachan, 1999; Macdonald & Harrington, 2003; Nordström et al., 2003; Banks et al., 2008). Interestingly, the escapee events may threaten native wild American mink populations in their natural range, through hybridization, outbreeding depression, and competition for food, space, and mates (Bowman et al., 2007).

Farmed American mink are usually housed in relatively high densities, which favours multiplication and spread of pathogens. Therefore, domestic mink which escape from farms can transmit Aleutian mink disease virus (AMDV) and other pathogens (*e.g.*, zoonotic *Cryptosporidium* sp.) that may seriously diminish reproduction and survival rate of feral mink populations (Nituch et al., 2011; Qian et al., 2020). *N. vison* is also reported to be susceptible to coronaviruses (Vlasova et al., 2011). Recently, American mink were identified as hosts of severe acute respiratory syndrome coronavirus 2 (SARS-CoV-2), the causative agent of deadly for humans COVID-19 disease (Hossain, Akter & Saha, 2020). The transmission of SARS-CoV-2 to American mink was confirmed in several countries (*e.g.*, in the Netherlands and Denmark), where increased mortality of animals was observed (Molenaar et al., 2020; Maron, 2020).

Among several disease conditions described for American mink, currently the most significant one is Aleutian mink disease (AMD), a highly pathogenic parvovirus affecting American mink and other mustelids (Bloom, Kanno & Mori, 1994; Virtanen et al., 2020). The AMD transmission is horizontal (*via* blood, saliva, faeces or urine) or vertical (seropositive dams during the perinatal period infects kits) (Alexandersen et al., 1985) and clinical symptoms in adult mink include hypergammaglobulinemia, kidneys glomeruli inflammation, reduced fertility, and spontaneous abortion (Alexandersen, 1986; Bloom, Kanno & Mori, 1994). Occasionally AMD leads to neurologic disease associated with a nonsuppurative meningoencephalitis (Jahns et al., 2010). Kits affected by AMD show rapidly progressing interstitial pneumonia, extensive atelectasis and hyaline membrane formation, which results in high mortality rates. Depending on strain, mortality rate varies from 30% to over 90%, and adult survivors develop typical lesions of adult form (Alexandersen, 1986).

AMD is caused by Aleutian mink disease virus, a single-stranded DNA parvovirus of the *Amdoparvovirus* genus (Cotmore et al., 2014). Similarly to other parvoviruses, AMDV replicates using rolling hairpin mechanism (Cotmorel & Tattersall, 1996). However, AMDV can be distinguished from other parvoviruses by a host response to exposure, which contributes to the progression of disease symptoms (Castelruiz, Blixenkrone-Moller & Aasted, 2005). The virus has a genome of approx. 4.8 kbp and non-enveloped virion contains two structural (VP1 and VP2) and three non-structural (NS1, NS2 and NS3) proteins (Bloom et al., 1988). Among these proteins NS1 and VP2 are suggested to have crucial roles in pathogenicity, since a high degree of variability in both amino acid sequences between highly virulent and non-virulent strains was recorded (Best, Wolfinbarger & Bloom, 2002). 

AMDV has frequently been reported in feral and wild American mink populations in Europe and North America. Yamaguchi & Macdonald (2001) found that 52% of investigated feral individuals (only adults) in the upper Thames region of the UK were anti-AMDV antibodies positive. In France, 23% of caught free-ranging American mink were infected with AMDV (Fournier-Chambrillon et al., 2004), while in a Spanish feral population 40% of tested individuals of *N. vison* were infected (Mañas et al., 2001). Wild populations were also tested in *N. vison*’s native range, *i.e.,* Nova Scotia (Canada), where 93% of collected samples were positive (Farid, 2013).

Isolated islands are interesting areas for virus studies. Jensen et al. (2012) showed high, up to 45%, occurrence of AMDV in feral American mink in Bornholm (Denmark). Additionally, virus strains were found to be clustered into two groups, suggesting introduction of Swedish and Danish strains to Bornholm (Jensen et al., 2012). In Iceland, samples from 1986–87 showed minimal (3.5%) prevalence of AMDV in the feral population (Skírnisson, Gunnarsson & Hjartardóttir, 1990). Moreover, Ryt-Hansen et al. (2017) proved that 4 sequences of partial NS1 gene from Icelandic free-ranging mink were close to the Swedish virus strain. 

Iceland, due to its insulated character, provides a unique opportunity to accurately monitor spread and persistence of pathogens in feral populations. Therefore, the overarching objective of this study was to assess the prevalence of AMDV in the feral population of American mink in Iceland. Additionally, we aimed to address the question whether AMDV-infected American mink in the wild exhibit deterioration in their health status.

## Materials & Methods

### Sample collection, morphometric analysis and tissue sampling

Feral mink were hunted by professional mink hunters using hunting dogs or death traps as part of an official control program funded by Icelandic authorities. A total of 164 (100 males and 64 females) American mink carcasses of mink caught in 2010–2018 in 8 regions were obtained for post-mortem analyses and sample collection performed at the West Iceland Nature Research Centre. Body weight to the nearest 1 g and total body length (excluding tail) to the nearest 0.5 cm were assessed and sex was determined. The entire spleen was removed and weighed to the nearest 0.01 g. Spleen samples were packed individually in sterile bags and stored at −20°C until delivery to the Faculty of Food Science and Fisheries of the West Pomeranian University of Technology (Szczecin) molecular laboratory for later DNA extraction and analysis.

### AMDV detection

Spleen samples were used for DNA extraction which was performed using the High Pure PCR Template Preparation Kit (Roche) according to the manufacturer’s instructions. The quantity and quality of DNA isolates were assessed using a NanoDrop 2000 (Thermo Scientific) and by separation in 1.5% agarose gel. The extracted DNA was tested using end-point PCR and unique set of primers to amplify 374 bp fragment of the AMDV NS1 gene: AMDV-F-7-H-PN1: 5′-CATATTCACTGTTGCTTAGGTTA-3′ and AMDV-R-7-HPN2: 5′-CGTTCTTTGTTAGTTAGGTTGTC-3′ (Jensen et al., 2011). Amplifications were conducted on Mastercycler (Eppendorf) under following conditions: 1 step of 5 min at 94 °C followed by 45 cycles at 94 °C for 30 s, 55 °C for 30 s, 72 °C for 30 s, and final extension at 72 °C for 7 min. Reactions were prepared with the GoTaq PCR kit (Promega), including 5 µl of Green GoTaq^®^  Flexi Buffer, 2.5 µl of MgCl_2_ (25 mM Solution), 0.5 µl of PCR Nucleotide Mix (10 mM), 0.125 µl of GoTaq^®^  DNA Polymerase (5 u/l), 0.5 µM of each primer and 3 µl of DNA template in final volume of 25 µl. Negative sample was applied to ensure specificity of PCR reaction. The results of end-point PCR reactions were assessed by 2.0% gel electrophoresis and positive samples sequenced on both strands by means of direct Sanger sequencing by the Genomed company (Warsaw) to eliminate false-positive results. Raw sequences were processed, aligned and checked using the sequence editing software Geneious v.11.1.5 (created by Biomatters, available from http://www.geneious.com). Additionally, all sequences were translated into amino acids to confirm their accuracy and to detect the presence of nuclear DNA pseudogenes, insertions, deletions or stop codons. Finally, all sequences were searched for matches against the GeneBank database using BLASTn and number of haplotypes were calculated using the DNAsp 6.0 software (Rozas et al., 2017).

### Condition index, statistical analysis and data visualization

**Figure 1:**
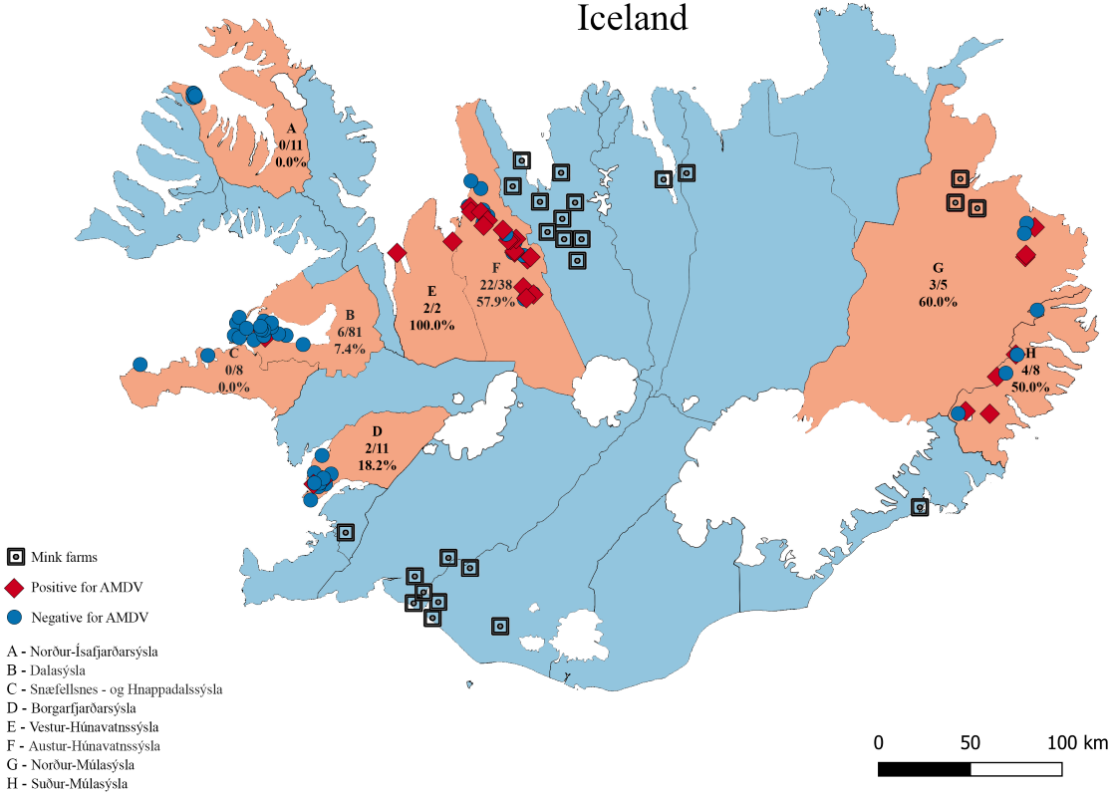
The eight sampling regions (light red) in Iceland. Sample sites are indicated with red diamonds (AMDV positive) and blue dots (AMDV negative). Mink farms in operation in 2012 are shown in black. The number of AMDV positive samples/total number of samples are indicated for each region.

Clear sexual dimorphism of examined morphometric parameters was observed. Therefore, Fulton’s condition factor (*K*) and the ratio of spleen weight to body weight were used for the comparison of the potential impact of viral infection. Fulton’s condition factor was calculated with the following formula: $K=100\times W\times {L}_{b}^{-3}$, where *K* is Fulton’s condition factor, *W* is body weight (g) and L_*b*_ is body length (cm). A Mann–Whitney U test was used to check significance of differences between Fulton’s condition indexes, spleen weight and body weight of infected and AMDV-free males and females and pairwise comparisons between groups were conducted to correct for multiple testing (post-hoc tests). All sampled individuals from across Iceland as was treated as exchangeable. The theoretical linear distance between sampling site and nearest mink farm was measured based on Stefansson, Schmalensee & Skorupski (2016) data (Fig. 1). That distance is likely to differ from a true distance travelled by feral mink in the area, since American mink migrate mainly along waterways and coastline (Macdonald et al., 2015). The simplification was implemented assuming the same systematic error for all the samples, which allows to correctly interpret the results. The Median test (Zar, 2010) was used to compare distance from sampling site to the nearest mink farm between AMDV-infected and virus-free individuals. The map was produced using the QGIS 3.12 software (QGIS Development Team, available from http://qgis.osgeo.org). Statistical analyses were performed using the Statistica software (Statsoft) and data were visualised using R version 4.0.3 (R Core Team, 2020) and R-package ggplot2 (Wickham, 2016).

## Results

### Virus detection

**Figure 2:**
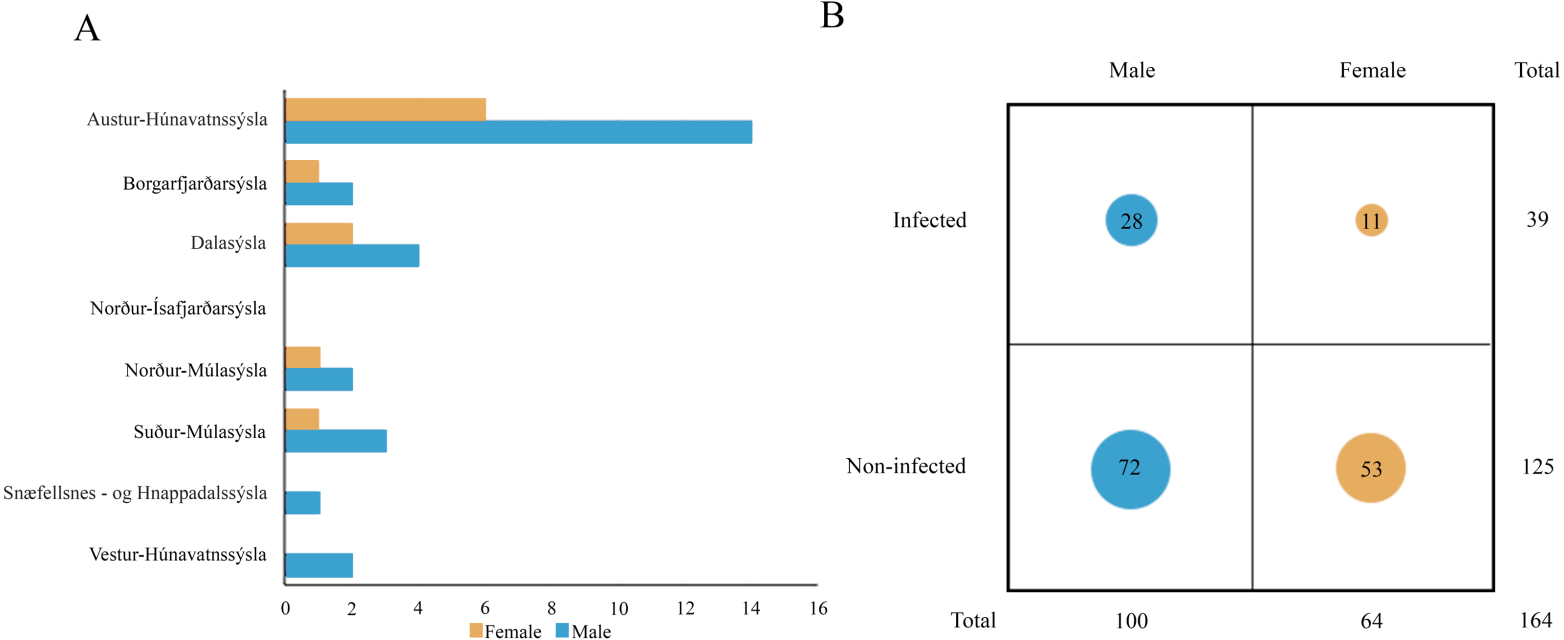
(A) Distribution of AMDV positive individuals (*n* = 39) collected in eight regions of Iceland in sex groups. (B) Comparison of AMDV infection rate (diameter of the circles) in both sexes. An analysis of Pearson’s Chi-squared test with Yates’ continuity correction (*χ*2 = 1.9559, *df* = 1, *p*-value = 0.162) indicated non-significant difference.

**Figure 3:**
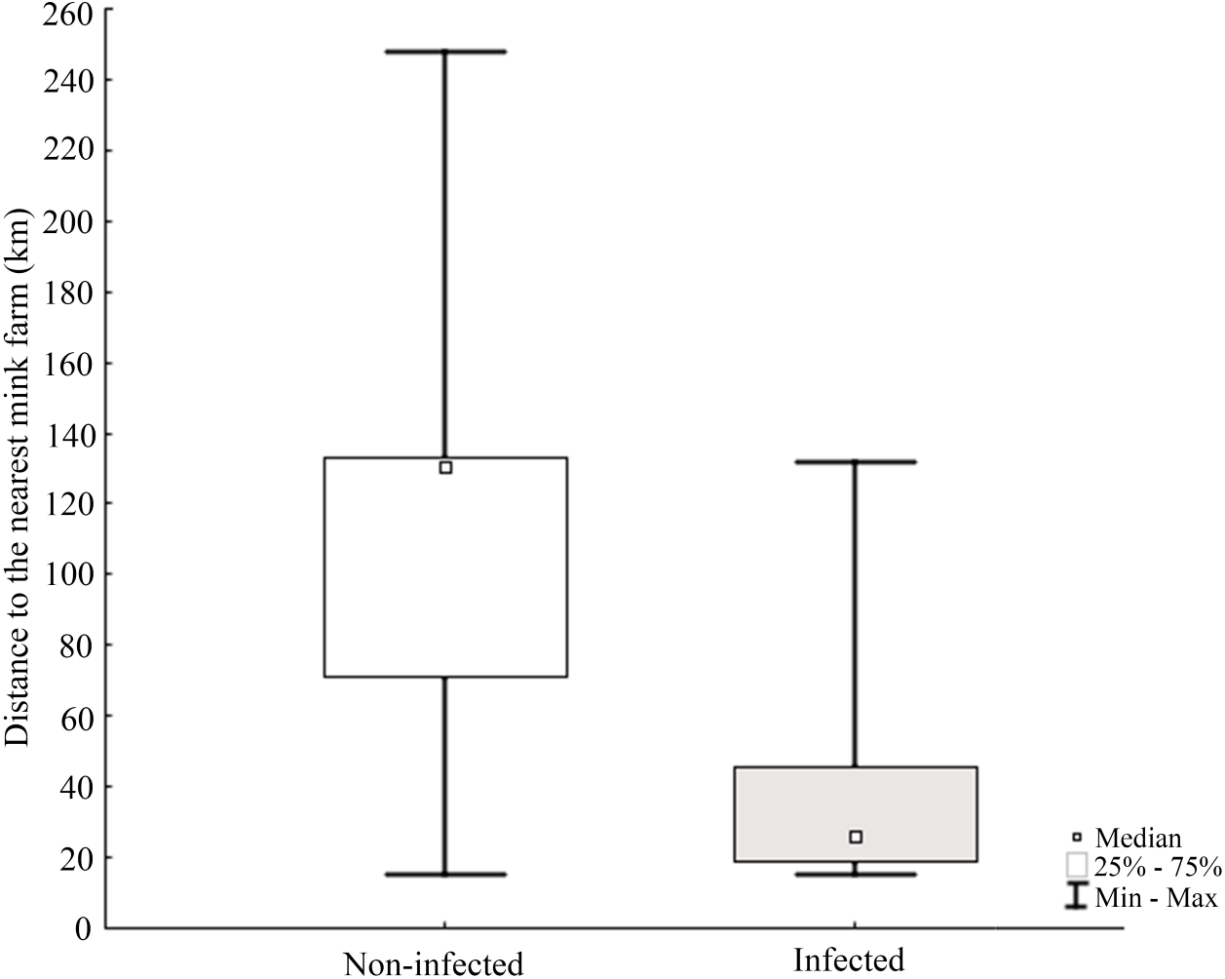
Comparison of distance between nearest farm and AMDV infected or non-infected American mink individuals. The Pearson’s Chi-squared test (*χ*2 = 29.388, *df* = 1, *p* = 0.0000) indicates a significant difference.

Molecular analysis showed that 23.8% of collected samples were AMDV positive (39 out of 164) and 33 haplotypes were identified using the DNAsp 6.0 software (Supplementary Information S1). AMDV haplotypes sequencing data are available at GenBank under accession numbers MW772385–MW772417. While most of the animals sampled in our study were males (Fig. 2A), Pearson’s χ2 test with Yates’ continuity correction revealed no significant difference in infection rate between males and females (Fig. 2B). Statistical analysis of distances of sample locations relative to mink farm location (Stefansson, Schmalensee & Skorupski, 2016) revealed that AMDV infected American mink were on average closer (40.8 km) to farms than disease free mink (117.4 km), (Figs. 1 and 3).

### Morphometry and condition index

**Figure 4:**
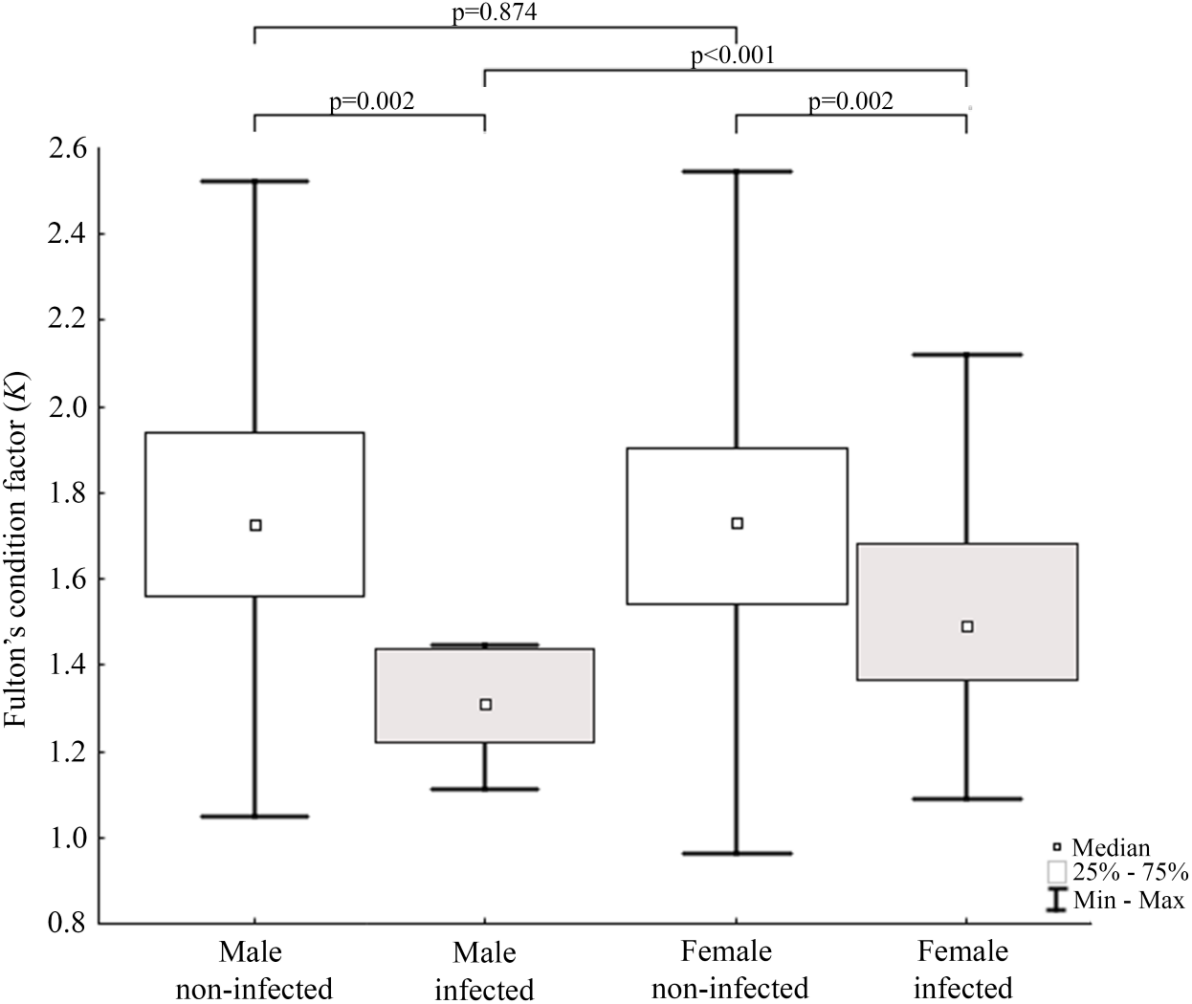
Evaluation of Fulton’s condition factor (*K*) for mink in Iceland using Mann–Whitney U test. Comparison of Fulton’s condition factor between specimens of mink: infected and non-infected males (*Z* = 3.183); infected and non-infected females (*Z* = 3.183); non-infected males and non-infected females (*Z* = −0.159); infected males and infected females (*Z* = 2.576).

**Figure 5:**
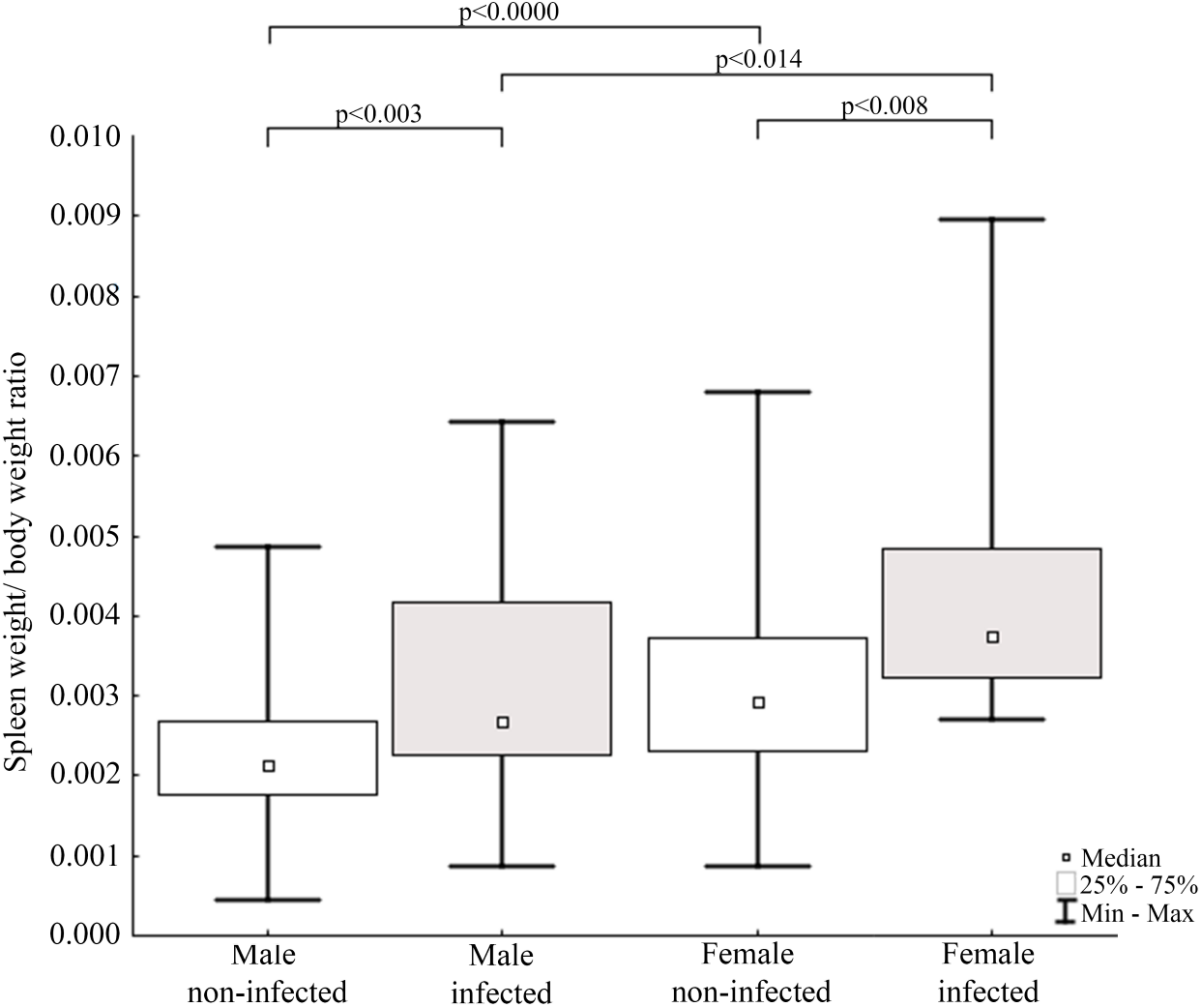
Evaluation of spleen weight/body weight ratio for mink in Iceland using Mann–Whitney U test. Comparisons of spleen weight/ body weight ratio between specimens of mink: infected and non-infected males (*Z* = −2.935), infected and non-infected females (*Z* = −2.586), non-infected males and non-infected females (Z = −3.915), infected males and infected females (*Z* = 2.403).

Results of statistical analysis of studied morphometric features revealed significantly higher body weight (*t*-test; = 11.653, *df* = 145; *p* < 0.0001) and body length (*t*-test; *t* = 15.186; *df* = 133; *p* < 0.001) of males comparing to females. Moreover, study showed low values of the variation coefficient (less than 10%) for the mean body length of both males and females (Table 1). Comparison of Fulton’s condition factor (*K*) between non-infected and infected individuals revealed significantly higher value of this indicator in non-infected males and females. Moreover, infected males had significantly lower *K* than infected females and now difference in *K* was found between AMDV-free males and females (Fig. 4). Additionally, the study revealed significantly higher spleen weight to body weight ratio in males and females infected with AMDV, and higher ratio in females comparing to males, regardless of the infection (Fig. 5).

## Discussion

### Virus detection

Isolated islands are unique places for virological studies, especially because of the limited influx of virus strains from the outside. Here, we confirmed presence of AMDV in free-ranging American mink in 6 (Austur-Húnavatnssýsla, Borgarfjar ðarsýsla, Dalasýsla, Nor ður-Múlasýsla, Su ður-Múlasýsla and Vestur-Húnavatnssýsla) out of 8 investigated regions (Fig. 1). AMDV was not detected in two regions (Nor ður-Ísafjar ðarsýsla, Snæfellsnes - og Hnappadalssýsla). However, to confirm our outcomes further research should focus on different disease tracking methods in such areas. For instance, non-invasive methods such as collecting faeces combined with a sensitive detection method, *i.e.,* qPCR (Virtanen et al., 2020). This approach has been successfully used for the detection of amdoparvoviruses in farmed American mink (Virtanen et al., 2020), raccoon dog (*Nyctereutes procyonoides*) and Arctic fox (*Vulpes lagopus*) (Shao et al., 2014) or wild red fox (*Vulpes vulpes*) (Bodewes et al., 2014).

**Table 1:** Basic morphometric parameters taken from all *N. vison* individuals.

	Mean		Standard deviation		Lower 95% conf. limit		Higher 95% conf. limit		Coef. of variation %	
Sex	Male	Female	Male	Female	Male	Female	Male	Female	Male	Female
Body length (excluding tail) (cm)	41.2	35.2	2.46	1.54	40.6	34.72	41.7	35.60	5.98	4.38
Body weight (g)	1173.3	728.9	258.99	136.53	1121.1	692.72	1225.5	765.17	22.07	18.73
Spleen weight (g)	2.9381	2.3922	1.4838	1.1086	2.6358	2.1058	3.2404	2.6785	50.50	46.34
Fulton’s condition factor *K*	1.67	1.67	0.3132	0.339	1.61	1.58	1.74	1.77	18.73	20.24
Spleen weight/body weight ratio	0.0026	0.0033	0.0012	0.0015	0.002	0.0029	0.003	0.0038	46.79	45.55

The study showed that 23.8% of collected mink samples were found AMDV positive and the result is consistent with a previous study of Ryt-Hansen et al. (2017), who reported AMDV in 25% (4 out of 16) of samples collected in Iceland, however, no information on sampling sites were provided. These results are similar to estimates in France where AMDV prevalence amounted to 23% (Fournier-Chambrillon et al., 2004), but lower than in other countries where the feral American mink is considered an invasive species, *i.e.,* Sweden (Persson et al., 2015), Denmark (Jensen et al., 2012), and Spain (Mañas et al., 2001), where AMDV prevalence amounted to 57%, 45% and 40% respectively. The dominance of male individuals (in collected and AMDV-positive samples) may be related to hunting methods used in this study, since they have been found to be more susceptible to trapping, because of wider home-range size (Buskirk & Lindstedt, 1989). Thus, considering similar infection rate of males and females, the main type of AMDV transmission in Icelandic free-ranging American mink remains unclear. According to Alexandersen et al. (1985) and Macdonald et al. (2015) horizontal transmission occurs mainly in males, due to roam-and-mate tactics, but vertical transmission from dams to kits during the perinatal period were equally plausible in both sexes. Additionally, our results contrast with the hypothesis that in mammals male individuals are more susceptible to pathogens, since revealed infection rate was similar for both males and females (Moore & Wilson, 2002).

The results indicated that AMDV infected American mink were on average closer to farms than disease free individuals. Nituch et al. (2011) reported similar findings with a higher prevalence of infected mink near farms in a large-scale study conducted in Canada, which indicated mink farms as a source of AMDV spread in the wild population of American mink. In some countries, such a distribution might be explained by newly escaped, infected farmed mink tending to occupy areas close to the farm they escaped from (Ionescu et al., 2018). Such an explanation is however highly unlikely in this study. Icelandic feral mink are distinctively different from farmed mink in body size and fur quality (Stefansson, Schmalensee & Skorupski, 2016), and farm escapees are thus very easily recognized. During the sampling from mink carcasses used in this study, every animal was assessed as a part of the feral population (pers. comm. Stefansson and von Schmalensee). It should be noted that in general, feral or wild American mink may also transmit pathogens to domestic mink, and at least one such scenario has been reported (Nituch et al., 2012). But the occurrence of pathogen transmission between free-ranging and domestic mink needs to be investigated further. The frequency of spill-back events may eventually depend on many various local factors, such as the infection rate in the free-ranging mink and their population density near mink farms, the biosecurity measures taken at farms but also natural behaviour during the mating season, since they display scramble competition polygyny *i.e.,* competitive mate search (Alcock, 1998).

This study sheds light on the prevalence of AMDV in parts of Iceland but does not provide conclusive evidence about the direction of AMDV transmission, as samples were not obtained throughout whole country, nor from mink farms. Further studies should address possible factors which may influence the main routes of AMDV transmission, especially historical background, specific for Iceland. When AMDV entered the feral mink population in this country in 1970–1983, the seropositive farms were all located in the northern part of the island. The highest proportion of seropositive feral mink in 1986-87 were also located in N-Iceland (Skírnisson, Gunnarsson & Hjartardóttir, 1990), and the highest infection rate in feral mink has continued to be connected to N-Iceland (Stefánsson et al., 2004; Stefánsson et al., 2011).

From the ecological point of view, the biggest concern in Iceland is the potential risk of AMDV transmission to Arctic fox (*Vulpes lagopus*), the only native carnivore in Iceland (Unnsteinsdottir et al., 2016). AMDV has been detected in red foxes (*Vulpes vulpes*), proving that AMDV is capable of infecting canids, but to assess the virus threat for *V. lagopus* further investigations are needed (Canuti et al., 2020a).

In light of the COVID-19 pandemic and reported circulation of SARS-CoV-2 virus between human and American mink (Maron, 2020) our results may indicate how virus can spillover to the natural environment in isolated islands, such as Iceland. However, from the early views the transmission of SARS-CoV-2 appears to be much more rapid (Molenaar et al., 2020) than AMDV, which is transmitted poorly and can be controlled in farm conditions (James & Dubovi, 2016). Therefore, sealing the American mink farms and minimization of escapees is critical to both stop viruses spread and avoid the need for radical moves, like culling of the entire populations in Netherlands and Denmark (Molenaar et al., 2020; Maron, 2020).

### Morphometry and condition index

The low values of the variation coefficient (less than 10%) for the mean body length of both males and females are an interesting aspect of the results. The high variability of mean values of other analysed parameters indicates an existence of a sex independent separating factor. Comparisons showed significantly higher body weight and body length of males comparing to females that is consistent with the sexual dimorphism in the species (*e.g.*, Kruska & Schreiber, 1999). Considering that the separating factor, mentioned above, does not differentiate individuals with respect to body length, but other morphometric indices studied, suggests that it is a pathogenic factor, possibly AMDV. This factor divides the studied population into at least two statistical subpopulations, within both males and females.

The observed sexual dimorphism is typical for this species (at all development stages) and has been previously reported elsewhere (Melero et al., 2012; Macdonald et al., 2015). Moreover, male and female American minks’ body length coefficient of variation indicates a homogeneous population (in statistical terms) relative to this feature. Assuming that body length is a relatively stable feature in adult organisms, high coefficient of variation values of other features indicates influence of differentiating factors. In further analysis we considered AMDV infection as such factor since its impact on *N. vison* health status and condition was previously reported (Eklund et al., 1968).

Condition indices are a simple method to investigate influence of external factors on an organism, and therefore its health status (Stevenson & Woods Jr, 2006). Fulton’s condition factor (*K*) is generally used in fishery studies, but the potential of its application in small mammals has been reported (Peig & Green, 2010). In the study non-infected mink had significantly higher value of *K* comparing to AMDV-infected individuals. Jensen, Chriél & Hansen (2015) showed worsening condition of American mink infected with AMDV in the experimental challenges. Our results showed similar tendency in free-ranging and adjacent to farms population of American mink, regardless of sex. The worse condition of infected *N. vison* individuals could be explained with symptoms (anaemia, anorexia) described for AMD (Eklund et al., 1968). In contrast, Canuti et al. (2020b) revealed no association between AMDV infection and body condition in Pacific marten (*Martes caurina*) and the North American river otter (*Lontra canadensis*), although it is unclear whether these species develop disease symptoms or act as asymptomatic carriers. Additionally, study found that only infected males had significantly lower *K* than infected females. Thus, indicating a stronger negative impact on males compared to females, despite similar infection rate. To our knowledge, no reports on the sexual disparities in response to AMDV infection have been published elsewhere, however during an American mink population decrease in Iceland (2004–2010) male individuals were significantly more impaired than females (Magnusdottir et al., 2014). An analogous pattern was reported in house mouse (*Mus musculus*) infected with influenza virus (Celestino et al., 2018), while male performed better than female in the South American coati (*Nasua nasua*) infected with trypanosomatids and gastrointestinal parasites (Olifiers et al., 2015).

The size of immune organs (*i.e.,* spleen, performing important immune functions) has been commonly used to describe animals’ investment in immunological response to various factors (John, 1994; Schulte-Hostedde, Bowman & Nituch, 2012). In the study AMDV infected mink had significantly higher spleen weight to body weight ratio. The enlargement of spleen was most likely caused by pathogen presence, which means immune organs were in a highly activated (primed) state and increased number of lymphocytes (*e.g.*, T-cells) were produced (Abolins et al., 2017). The spleen enlargement of AMDV infected mink is consistent with previous findings (Reichert & Kostro, 2014). Our results were also comparable to findings of Persson et al. (2015), where difference in relative spleen weight was found in male free-ranging American mink, but no difference was found in females, probably due to a smaller sample size. Additionally, regardless of the infection females had higher spleen weight to body weight ratio than males. The difference of relative spleen weight between sexes has been reported elsewhere (Schulte-Hostedde, Bowman & Nituch, 2012; Persson et al., 2015), but the reason for sexual disparity remains unclear. Moore & Wilson (2002) suggested a shift of energy resources towards reproduction and/or growth over the immune system in male mammals and immunosuppressive side effects of androgenic hormones. While Nunn et al. (2009) found no differences in immune system functioning associated with sexual dimorphism. Therefore, males’ lower value of relative spleen weight reported in our study should be further investigated. Moreover, we propose Fulton’s condition factor combined with spleen weight to body weight ratio to be a preliminary measure to conduct further testing for AMDV infection in American mink using combination of more sensitive methods.

## Conclusions

The results of our study provide a simple method to preliminarily assess the possibility of an AMDV infection based on simple and quick body measurements of American mink. The reported distribution and prevalence of AMDV may be used as starting point for further studies on prevalence and transmission of different pathogens (*e.g.*, SARS-CoV-2, *Cryptosporidium* sp.) in Iceland and other isolated ecosystems. Of particular importance is to study feral and wild American mink as potential reservoirs of SARS-CoV-2, as possibility of infection to American mink has been proved (Molenaar et al., 2020; Maron, 2020). Further studies should focus on assessment of AMDV prevalence in the remaining counties in Iceland and identification of other pathogens that might be transmitted in a latent form by heathy animals. In general, monitoring of diseases in free-ranging populations in the vicinity of mink farms should be commenced world-wide, to evaluate both the prevalence of pathogen spill-over from farms and the risk of pathogen spill-back from feral or wild populations.

## Supplemental Information

10.7717/peerj.12060/supp-1 Supplemental Information 1 A. The detection of AMDV in the spleen of male and female individuals of American mink (*n* = 164) in eight regions of Iceland. B. Morphometric GPS data

10.7717/peerj.12060/supp-2 Supplemental Information 2 Sequence data of Aleutian mink disease virus (AMDV) Sequences have been deposited in GenBank, but they are not publicly yet (access 09/21/2021).

## Additional Information and Declarations

### Data Availability

The following information was supplied regarding data availability: The raw data are available in the Supplementary File. The sequences are available in the Supplemental File and at GenBank: MW772385–MW772417.
