# Supplementary material for: Assessment of Aleutian mink disease virus (AMDV) prevalence in feral American mink in Iceland. Case study of a pending epizootiological concern in Europe
Source: PeerJ. 2021 Sep 17;9:e12060. doi: 10.7717/peerj.12060 (PMC8451439; doi:10.7717/peerj.12060)
Supplement: Supplemental Information 2 — Sequences have been deposited in GenBank, but they are not publicly yet (access 09/21/2021). [file peerj-09-12060-s002.pdf]

Genbank Accesion number	AMDV isolate
MW772385	IS_H1_R3213-R3214-R3215-R3216
MW772386	IS_H2_R3232
MW772387	IS_H3_R3233
MW772388	IS_H4_R3236
MW772389	IS_H5_R3237
MW772390	IS_H6_R3238
MW772391	IS_H7_R3240
MW772392	IS_H8_R3241
MW772393	IS_H9_R3243-R3245
MW772394	IS_H10_R3244
MW772395	IS_H11_R3301-R3340
MW772396	IS_H12_R3317-R3318
MW772397	IS_H13_R3335
MW772398	IS_H14_R3336
MW772399	IS_H15_R3337
MW772400	IS_H16_R3341
MW772401	IS_H17_R3348
MW772402	IS_H18_R3349
MW772403	IS_H19_R3351
MW772404	IS_H20_R3353
MW772405	IS_H21_R3354
MW772406	IS_H22_R3355
MW772407	IS_H23_R3358
MW772408	IS_H24_R3359
MW772409	IS_H25_R3360
MW772410	IS_H26_R3361
MW772411	IS_H27_R3362
MW772412	IS_H28_R3365
MW772413	IS_H29_R3366
MW772414	IS_H30_R3367
MW772415	IS_H31_R3370
MW772416	IS_H32_R3371
MW772417	IS_H33_R3372

>Seq1 [organism=organism=Aleutian mink disease virus] Aleutian mink disease virus isolate AMDV_IS_H1 non-structural protein 1 gene (NS1), partial cds
CTTTGATAAAGATGAAGATCCTAAGGATATTCAAAAATCCTTAGGTTGGTTTATT
AAAAAACTAAATAGAGACATAGCAGTTGTTTATAGTAACCATCATTGTGACATAC
AAGGTATACAAGATCCTGAAAGTAGATCTAATAACTTGAAAGTATGGATTGAAG
ATGGGCCTACTAAACCTTACAAATACTTTAACAAACAAACCAAACAAGAATACA
ACAAACCAGTGAACATACGTGACTATACATTAATATATCTGTTTAACAAAGATAA
GATAACAGAAGAAGGTATGGATGGTTACTATGCTAGTGGTAACGGCGGCATTAT
T

>Seq2 [organism=organism=Aleutian mink disease virus] Aleutian mink disease virus isolate AMDV_IS_H2 non-structural protein 1 gene (NS1), partial cds
CTTTGATAAAGATGAAGATCCTAAGGATATTTAAAAATCTTTAGGTTGGTTTATT
AAAAAACTAAGTAAAGATATAGCAGTTGTTTATAGTAACCATCATTGTGACATGC
AAGGTATACAAGATTCTGAAAGTAGATCTAATAACTTGAAAATATGGATTGAAG
ATGGACCTACCAAACCTTACAAATACTTTAACAAACAAACCAAACAAGAATACA
ACAAACCAGTGAACATACGTGACTATACATTAATATATCTGTTTAACAAAGATAA
GATAACAGAGGAAGGTATGGATGGTTACTATGCTAGTGGTAACGGCGGCATTAT
T
>Seq3 [organism=organism=Aleutian mink disease virus] Aleutian mink disease virus isolate AMDV_IS_H3 non-structural protein 1 gene (NS1), partial cds
CTTTGATAAAGATGAAGATCCTAAGGATATTCAAAAATCCTTAGGTTGGTTTGTT
AAAAAACTAAATAAAGACTTAGCAGTTGTTTATAGTAACCATCATTGTGACATAC
AAGGTATACAAGATCCTGAAAGTAGATCTAATAACTTGAAAATATGGATTGAAG
ATGGACCTACCAAACCTTACAAATACTTTAACAGACAAACCAAACAAGAATACA
ACAAACCAGTGAACATGCGTGACTATACATTAATATATCTGTTTAACAAAGATAA
AATAACAGAAGAAAGTATGGTTGGTTACTATGCTAGTGGTAACGGTGGCATTATT
>Seq4 [organism=organism=Aleutian mink disease virus] Aleutian mink disease virus isolate AMDV_IS_H4 non-structural protein 1 gene (NS1), partial cds
CTTTAATAAAGATGAAGATCCTAAGGATATTCAAAAATCCTTAGGTTGGTTTATT
AAAAAACTAAGTAAAGACTTAGCAGTTGTTTATAGTAACCATCATTGTGACCTGC
AAGGTATACAAGACTCTGAAAGTAGATCTAATAACTTAAAAGTATGGATTGAAG
ATGGGCCTACAAAACCTTACAAATACTTTAACAGACAAACCAAACAAGAATACA
ACAAACCAGTAAACATACGTGACTATACAATAGTATATCTGTTTAACAAAGATA
AGATAACAGAAGAAGGTATGGATGGTTACTATGCTAGTGGTAACGGCGGCTTTA
TT
>Seq5 [organism=organism=Aleutian mink disease virus] Aleutian mink disease virus isolate AMDV_IS_H5 non-structural protein 1 gene (NS1), partial cds
CTTTAATAAAGATGAAGATCCTAAGGATATTCAAAAATCCTTAGGTTGGTTTATT
AAAAAACTAAGTAAAGACATAGCAGTTGTTTATAGTAACCATCATTGTGACCTGC
AAGGTATACAAGACTCTGAAAGTAGATCTAATAACTTGAAAGTATGGATTGAAG
ATGGACCTACAAAACCTTACAAATACTTTAACAAACAAACCAAACAAGAATACA
ACAAACCAGTGAACATACGTGACTATACATGTGTATATCTGTTTAACAAAGATAA
GATAACAGAAGAAGGTATGAATGGTTACTATGCTAGTGGTAACGGCGGCATTAT
T
>Seq6 [organism=organism=Aleutian mink disease virus] Aleutian mink disease virus isolate AMDV_IS_H6 non-structural protein 1 gene (NS1), partial cds
CTTTGATAAAGATGAAGATCCTAAGGATATTCAAAAATCCTTAGGTTGGTTTGTT
AAAAAACTAAGTAGAGACATAGCAGTTGTTTATAGTAACCATCATTGTGACCTGC
AAGGTATACAAGATTCTGAAGGTAGATCTAATAACTTGAAAGTATGGATTGAAG
ATGGACCTACAAAACCTTACAAATACTTTAACAAACAAACCAAACAAGAATACA
ACAAACCAGTGAACATACGTGACTATACATGTGTATATCTGTTTAACAAAGATAA
GATAACAGAAGAAGGTATGGATGGTTACTATGCTAGTGGTAACGGTGGCATTAT
T
>Seq7 [organism=organism=Aleutian mink disease virus] Aleutian mink disease virus isolate AMDV_IS_H7 non-structural protein 1 gene (NS1), partial cds
CTTTGATAAAGATGAAGATCCTAAGGATATTCAAAAATCCTTAGGTTGGTTTGTT
AAAAAACTAAGTAGAGACATAGCAGTTGTTTATAGTAACCATCATTGTGACCTGC
AAGGTATACAAGATTCTGAAGGTAGATCTAATAACTTGAAAGTATGGATTGAAG
ATGGACCTACCAAACCTTACAAATACTTTAACAAACAAACCAAACAAGAATACA

ACAAACCAGTGAACATACGTGACTATACATGTGTATATCTGTTTAACAAAGATAA
GATAACAGAAGAAGGTATGGATGGTTACTATGCTAGTGGTAACGGCGGCATTAT
T
>Seq8 [organism=organism=Aleutian mink disease virus] Aleutian mink disease virus isolate AMDV_IS_H8 non-structural protein 1 gene (NS1), partial cds
CTTTGATAAAGATGAAGATCCTAAGGATATTCAAAAATCCTTAGGTTGGTTTATT
AAAAAACTAAGTAGAGACATAGCAGTTGTTTATAGTAACCATCATTGTGACCTGC
AAGGTATACAAGATTCTGAAGGTAGATCTAATAACTTGAAAGTATGGATTGAAG
ATGGACCTACCAAACCTTACAAATACTTTAACAAACAAACCAAACAAGAATACA
ACAAACCAGTGAACATACGTGACTATACATGTGTATATCTGTTTAACAAAAATAA
GATAACAGAAGAAGGTATGGATGGTTACTATGCTAGTGGTAACGGCGGCATTAT
T
>Seq9 [organism=organism=Aleutian mink disease virus] Aleutian mink disease virus isolate AMDV_IS_H9 non-structural protein 1 gene (NS1), partial cds
CTTTAATAAAGATGAAGATCCTAAGGATATTCAAAAATCCTTAGGTTGGTTTATT
AAAAAACTAAGTAAAGACTTAGCAGTTGTTTATAGTAACCATCATTGTGACCTGC
AAGGTATACAAGACTCTGAAAGTAGATCTAATAACTTGAAAGTATGGATTGAAG
ATGGACCTACAAAACCTTACAAATACTTTAACAAACAAACCAAACAAGAATACA
ACAAACCAGTGAACATACGTGACTATACATGTGTATATCTGTTTAACAAAGATAA
GATAACAGAAGAAGGTATGGATGGTTACTATGCTAGTGGTAACGGCGGCATTAT
T
>Seq10 [organism=organism=Aleutian mink disease virus] Aleutian mink disease virus isolate AMDV_IS_H10 non-structural protein 1 gene (NS1), partial cds
CTTTAATAAAGATGAAGATCCTAAGGATATTCAAAAATCCTTAGGTTGGTTTGTT
AAAAAACTAAGTAAAGACATAGCAGTTGTTTATAGTAACCATCATTGTGACCTGC
AAGGTATACAAGATTCTGAAGGTAGATCTAATAACTTGAAAGTATGGATTGAAG
ATGGACCTACCAAACCTTACAAATACTTTAACAAACAAACCAAACAAGAATACA
ACAAACCAGTGAACATACGTGACTATACATGTGTATATCTGTTTAACAAAGATAA
GATAACAGAAGAAGGTATGGATGGTTACTATGCTAGTGGTAACGGCGGCATTAT
T
>Seq11 [organism=organism=Aleutian mink disease virus] Aleutian mink disease virus isolate AMDV_IS_H11 non-structural protein 1 gene (NS1), partial cds
CTTTGATAAAGATGAAGATCCTAAGGATATTCAAAAATCCTTAGGTTGGTTTATT
AAAAAACTAAGTAGAGACATAGCAGTTGTTTATAGTAACCATCATTGTGACCTGC
AAGGTATACAAGATTCTGAAAGTAGATCTAATAACTTGAAAGTATGGATTGAAG
ATGGACCTACCAAACCTTACAAATACTTTAACAAACAAACCAAACAAGAATACA
ACAAACCAGTAAACATACGTGACTATACATTAATATATCTGTTTAACAAAGATAA
GATAACAGAAGAAGGTATGGATGGTTACTATGCTAGTGGTAACGGCGGCATTAT
T
>Seq12 [organism=organism=Aleutian mink disease virus] Aleutian mink disease virus isolate AMDV_IS_H12 non-structural protein 1 gene (NS1), partial cds
CTTTGATAAAGATGAAGATCCTAAGGATGTTCAAAAATCCTTAGGTTGGTTTATT
AAAAAACTAAATAGAGACATAGCAGTTGTTTATAGTAACCATCATTGTGACATAC
AAGATATACAAGATCCTGAAAGTAGATCTAATAACTTGAAAGTATGGATTGAAG
ATGGGCCTACTAAACCTTACAAATACTTTAACAAACAAACCAAACAAGAATACA
ACAAACCAGTGAACATGCGTGACTATACAATAATATATCTGTTTAACAAAGATA
AGATAACAGAAGAAGGTATGGATGGTTACTATGCTAGTGGTAACGGCGGCATTA
TT
>Seq13 [organism=organism=Aleutian mink disease virus] Aleutian mink disease virus isolate AMDV_IS_H13 non-structural protein 1 gene (NS1), partial cds

CTTTGATAAAGATGAAGATCCTAAGGATATTCAAAAATCCTTAGGTTGGTTCATT
AAAAAACTAAATAAAGACATAGCAGTTGTTTATAGTAACCATCATTGTGACATGC
AAGGTCTACAAGATCCTGAAAGTAGATCTAATAACTTGAAAGTATGGATTGAAG
ATGGACCTACCAAACCTTACAAATACTTTAACAGACAAACCAAACAAGAATACA
ACAAACCAGTGAACATGCGTGACTATACATTAATATATCTGTTTAACAAAGATAA
GATAACAGAAGAAAGTATGGATGGTTACTATGCTAGTGGTAACGGTGGCATTAT
T
>Seq14 [organism=organism=Aleutian mink disease virus] Aleutian mink disease virus isolate AMDV_IS_H14 non-structural protein 1 gene (NS1), partial cds
CTTTGATAAAGATGAAGATCCTAAGGATATTCAAAAATCCTTAGGTTGGTTTATT
AAAAAACTAAATAAAGACATAGCAGTTGTTTATAGTAACCATCATTGTGACATGC
AAGGTATACAAGATCCTGAAAGTAGATCTAATAACTTAAAAGTATGGATTGAAG
ATGGACCTACCAAACCTTACAAATACTTTAACAGACAAACCAAACAAGAATACA
ACAAACCAGTGAACTTGCGTGACTATACATTAATATATCTGTTTAACAAAAATAA
GATAACAGAAGATGGTATGGATGGTTACTATGCTAGTGGTAACGGTGGCATTATT
>Seq15 [organism=organism=Aleutian mink disease virus] Aleutian mink disease virus isolate AMDV_IS_H15 non-structural protein 1 gene (NS1), partial cds
CTTTGATAAAGATGAAGATCCTAAGGATATTCAAAAATCCTTAGGTTGGTTTATT
AAAAAACTAAATAAAGACGTAGCAGTTGTTTATAGTAACCATCATTGTGACATGC
AAGGTATACAAGATCCTGAAAGTAGATCTAATAACTTGAAAATATGGATTGAAG
ATGGACCTACCAAACCTTACAAATACTTTAACAAACAAACCAAACAAGAATACA
ACAAAACAGTGAACATGCGTGACTATACATTAATATATCTGTTTAACAAAAATAA
GATAACAGAAGAAGGTATGGATGGTTACTATGCTAGTGGTAACGGTGGCATTAT
T
>Seq16 [organism=organism=Aleutian mink disease virus] Aleutian mink disease virus isolate AMDV_IS_H16 non-structural protein 1 gene (NS1), partial cds
CTTTGATAAAGATGAAGATCTTAAGGATATTCAAAAATCCTTAGGTTGGTTTGTT
AAAAAAATAAGTAGAGACATAGCAGTTGTTTATAGTAACCATCATTGTGACCTGC
AAGGTATACAAGATTTTGAAAGTAGATTTAATAACTTGAAAGTATGGATTGAAG
ATGGACCTTCCCAACCTTACAAATACTTTAACAACCAAACCAAACAAGAATACA
ACAACCCAGTGAACATACGTGACTATACATTAATATATCTGTTTAACAAAGATAA
GATAACAGAAGAAGGTATGGATGGTTACTATGCTAGTGGTAACGGCGGCATTAT
T
>Seq17 [organism=organism=Aleutian mink disease virus] Aleutian mink disease virus isolate AMDV_IS_H17 non-structural protein 1 gene (NS1), partial cds
CTTTGATAAAGATGAAGATCCTAAGGATATTCAAAAATCCTTAGGTTGGTTTATT
AAAAAACTAAATAGAGACATAGCAGTTGTTTTTAGTAACCATCATTGTGACATAC
AAGATATACAAGATCCTGAAGGTAGATCTAATAACTTGAAAGTATGGATTGAAG
ATGGGCCTACTAAACCTTACAAATACTTTAACAGACAAACCAAACAAGAATACA
ACAAACCAGTGAACATACGTGACTATACGTTCATATATCTGTTTAACAAAGATAA
GATAACAGAAGAAGGTATGGATGGTTACTATGCTAGTGGTAACGGCGGCATTAT
T
>Seq18 [organism=organism="Aleutian mink disease virus] Aleutian mink disease virus isolate AMDV_IS_H18 non-structural protein 1 gene (NS1), partial cds
CTTTGATAAAGATGAAGATCCTAAGGATATTCAAAAATCCTTAGGTTGGTTTATT
AAAAAACTAAATAGAGACATAGCAGTTGTTTTTAGTAACCATCATTGTGACATAC
AAGGTATACAAGATCCTGAAAGTAGATCTAATAACTTGAAAGTATGGATTGAAG
ATGGGCCTACTAAACCTTACAAATACTTTAACAAACAAACCAAACAAGAATACA
ACAAACCAGTGAACATACGTGACTATACATTAATATATCTGTTTAACAAAGATAA

GATAACAGAAGAAGGTATGGATGGTTACTATGCTAGTGGTAACGGCGGCATTAT
T
>Seq19 [organism=organism=Aleutian mink disease virus] Aleutian mink disease virus isolate AMDV_IS_H19 non-structural protein 1 gene (NS1), partial cds
CTTTGATAAAGATGAAGATCCTAAGGATATTCAAAAATCCTTAGGTTGGTTTATT
AAAAAACTAAGTAAAGACATAGCAGTTGTTTATAGTAACCATCATTGTGACATGC
AAGGTATACAAGATCCTGAAGGTAGATCTAATAACTTGAAAGTATGGATTGAAG
ATGGACCTACCAAACCTTACAAATACTTTAACAAACAAACCAAACAAGAATACA
ACAAACCAGTGAACATGCGTGACTATACATTAATATATCTGTTTAACAAAGATAA
GATAACAGAAGAAAGTATGGATGGTTACTATGCTAGTGGTAACGGTGGCATTAT
T
>Seq20 [organism=organism=Aleutian mink disease virus] Aleutian mink disease virus isolate AMDV_IS_H20 non-structural protein 1 gene (NS1), partial cds
CTTTGATAAAGATGAAGATCCTAAGGATATTCAAAAATCATTAGGTTGGTTTATT
AAAAAACTAAATAAAGACATAGCAGTTGTTTATAGTAACCATCATTGTGACATGC
AAGATATACAAGATCCTGAAGGTAGATCTAATAACTTGAAAGTATGGATTGAAG
ATGGACCAACCAAACCTTACAAATACTTTAACAGACAAACCAAACAAGAATACA
ACAAACCAGTGAACATGCGTGACTATACATTAATATATCTGTTTAACAAAAATAA
GATAACAGAGGAAGGTATGGATGGTTACTATGCTAGTGGTAACGGTGGCATTAT
T
>Seq21 [organism=organism=Aleutian mink disease virus] Aleutian mink disease virus isolate AMDV_IS_H21 non-structural protein 1 gene (NS1), partial cds
CTTTGATAAAGATGAAGATCCTAAGGATATTCAAAAATCCTTAGGTTGGTTTATT
AAAAAACTAAATAAAGACATAGCAGTTGTTTATAGTAACCATCATTGTGACATGC
AAGGTATACAAGATCCTGAAAGTAGATCTAATAACTTGAAAGTATGGATTGAAG
ATGGACCTACCAAACCTTACAAATACTTTAACAGACAAACCAAACAAGAATACA
ACAAACCAGTGAACATGCGTGACTATACATTAATATATCTGTTTAACAAAGATAA
GATAACAGAAGAAGGTATGAATGGTTACTATGCTAGTGGTAACGGTGGCATTAT
T
>Seq22 [organism=organism=Aleutian mink disease virus] Aleutian mink disease virus isolate AMDV_IS_H22 non-structural protein 1 gene (NS1), partial cds
CTTTGATAAAGATGAAGATCCTAAGGATATTCAAAAATCCTTAGGTTGGTTTATT
AAAAAACTAAATAAAGACATAGCAGTTGTTTATAGTAACCATCATTGTGACATGC
AAGGTATACAAGATCCTGAAAGTAGATCTAATAACTTGAAAGTATGGATTGAAG
ATGGACCTACCAAACCTTACAAATACTTTAACAAACAAACCAAACAAGAATACA
ACAAACCAGTGAACATGCGTGACTATACATTAATATATCTGTTTAACAAAAATAA
GATAACAGAAGATAGTATGGATGGTTACTATGCTAGTGGTAACGGTGGCATTATT
>Seq23 [organism=organism=Aleutian mink disease virus] Aleutian mink disease virus isolate AMDV_IS_H23 non-structural protein 1 gene (NS1), partial cds
CTTTGATAAAGATGAAGATCCTAAGGATATTCAAAAATCCTTAGGTTGGTTTATT
AAAAAACTAAATAAAGACTTAGCAGTTGTTTATAGTAACCATCATTGTGACCTGC
AAGGTATACAAGATACTGAAGGTAGATCTAATAATTTGAAAGTATGGATTGAAG
ATGGACCTACCAAACCTTACAAATACTTTAACAAACAAACCAAACAAGAATACA
ACAAACCAGTGAACATACGTGAATATACATTAATATATCTGTTTAACAAAGATAA
GATAACAGAGGAAGGTATGGATGGTTACTATGCTAGTGGTAACGGCGGCATTAT
T
>Seq24 [organism=organism=Aleutian mink disease virus] Aleutian mink disease virus isolate AMDV_IS_H24 non-structural protein 1 gene (NS1), partial cds
CTTTGATAAAGATGAAGATCCTAAGGATATTCAAAAATCCTTAGGTTGGTTTATT
AAAAAACTAAGTAGAGACTTAGCAGTTGTTTATAGTAACCATCATTGTGACATGC

AAGGTATACAAGATTCTGAAAGTAGATCTAATAACTTGAAAGTATGGATTGAAG
ATGGACCTACCAAACCTTACAAATACTTTAACAAACAAACCAAACAAGAATACA
ACAAACCAGTGAACGTACGTGACTATACAGTAGTATATCTGTTTAACAAAGATA
AGATAACAGAAGAAGGTATGGATGGTTACTATGCTAGTGGTAACGGCGGCATTA
TT
>Seq25 [organism=organism=Aleutian mink disease virus] Aleutian mink disease virus isolate AMDV_IS_H25 non-structural protein 1 gene (NS1), partial cds
CTTTGATAAAGATGAAGATCCTAAGGATATTCAAAAATCCTTAGGTTGGTTTATT
AAAAAACTAAATAGAGACTTAGCAGTTGTTTATAGTAACCATCATTGTGACCTGC
AAGGTATACAAGACTCTGAAAGTAGATCTAATAACTTGAAAGTATGGATTGAAG
ATGGGCCTACAAAACCTTACAAATACTTTAACAGACAAACCAAACAAGAATACA
ACAAACCAGTAAACATGCGTGACTATACAATAGTATATCTGTTTAACAAAGATA
AGATAACAGAAGAAGGTATGGATGGTTACTATGCTAGTGGTAACGGCGGCATTA
TT
>Seq26 [organism=organism=Aleutian mink disease virus] Aleutian mink disease virus isolate AMDV_IS_H26 non-structural protein 1 gene (NS1), partial cds
CTTTGATAAAGATGAAGATCCTAAGGATATTCAAAAATCCTTAGGTTGGTTTATT
AAAAAACTAAGTAAAGACTTAGCAGTTGTTTATAGTAACCATCATTGTGACCTGC
AAGGTATACAAGACTCTGAAAGTAGATCTAATAACTTGAAAGTATGGATTGAAG
ATGGGCCTACAAAACCTTACAAATACTTTAACAGACAAACCAAACAAGAATACA
ACAAACCAGTAAACATACGTGACTATACAGTAGTATATCTGTTTAACAAAGATA
AGATAACAGAAGAAGGTATGGATGGTTACTATGCTAGTGGTAACGGCGGCATTA
TT
>Seq27 [organism=organism=Aleutian mink disease virus] Aleutian mink disease virus isolate AMDV_IS_H27 non-structural protein 1 gene (NS1), partial cds
CTTTGATAAAGATGAAGATCCTAAGGATATTCAAAAATCCTTAGGTTGGTTTATT
AAAAAACTAAATAAAGACTTAGCAGTTGTTTATAGTAACCATCATTGTGACCTGC
AAGGTATACAAGACTCTGAAAGTAGATCTAATAACTTGAAAGTATGGATTGAAG
ATGGGCCTACAAAACCTTACAAATACTTTAACAGACAAACCAAACAAGAATACA
ACAAACCAGTAAACATACGTGACTATACAGTAGTATATCTGTTTAACAAAGATA
AGATAACAGAAGAAGGTATGGATGGTTACTATGCTAGTGGAACCGGCGGCTTTA
TT
>Seq28 [organism=organism=Aleutian mink disease virus] Aleutian mink disease virus isolate AMDV_IS_H28 non-structural protein 1 gene (NS1), partial cds
CTTTGATAAAGATGAAGATCCTAAGGATATTCAAAAATCCTTAGGTTGGTTTATT
AAAAAACTAAGTAGAGACTTAGCAGTTGTTTATAGTAACCATCATTGTGACATGC
AAGGTATACAAGATTCTGAAAGTAGATCTAATAACTTGAAAGTATGGATTGAAG
ATGGACCTACCAAACCTTACAAATACTTTAACAAACAAACCAAACAAGAATACA
ACAAACCAGTGAACGTACGTGACTATACATTAATATATCTGTTTAACAAAGATAA
GATAACAGAAGAAGGTATGGATGGTTACTATGCTAGTGGTAACGGCGGCATTAT
T
>Seq29 [organism=organism=Aleutian mink disease virus] Aleutian mink disease virus isolate AMDV_IS_H29 non-structural protein 1 gene (NS1), partial cds
CTTTGATAAAGATGAAGATCCTAAGGATATTCAAAAATCCTTAGGTTGGTTTATT
AAAAAACTAAATAGAGACATAGCAGTTGTTTATAGTAACCATCATTGTGACCTGC
AAGGTATACAAGATTCTGAAGGTAGATCTAATAACTTGAAAATATGGATTGAAG
ATGGACCTACCAAACCTTACAAATACTTTAACAAACAAACCAAACAAGAATACA
ACAAACCAGTGAACATGCGTGACTATACATTAATATATCTGTTTAACAAAGATAA
AATAACAGAAGAAGGTATGGATGGTTACTATGCTAGTGGTAACGGTGGCATTAT
T

>Seq30 [organism=organism=Aleutian mink disease virus] Aleutian mink disease virus isolate AMDV_IS_H30 non-structural protein 1 gene (NS1), partial cds
CTTTGATAAAGATGAAGATCCTAAGGATATTCAAAAATCCTTAGGTTGGTTTATT
AAAAAACTAAATAAAGACTTAGCAGTTGTTTATAGTAACCATCATTGTGACCTGC
AAGGTATACAAGACTCTGAAAGTAGATCTAATAACTTGAAAGTATGGATTGAAG
ATGGGCCTACAAAACCTTACAAATACTTTAACAGACAAACCAAACAAGAATACA
ACAAACCAGTGAACATACGTGACTATACATTAATATATCTGTTTAACAAAGATAA
GATAACAGAAGAAGGTATGGATGGTTACTATGCTGGTGGTAACGGCGGCATTAT
T
>Seq31 [organism=organism=Aleutian mink disease virus] Aleutian mink disease virus isolate AMDV_IS_H31 non-structural protein 1 gene (NS1), partial cds
CTTTGATAAAGATGAAGATCCTAAGGATATTCAAAAATCCTTAGGTTGGTTTATT
AAAAAACTAAGTAGAGACTTAGCAGTTGTTTATAGTAACCATCATTGTGACATGC
AAGATATACAAGATTCTGAAAGTAGATCTAATAACTTGAAAGTATGGATTGAAG
ATGGACCTACCAAACCTTACAAATACTTTAACAGACAAACCAAACAAGAATACA
ACAAACCAGTGAACATACGTGACTATACATTAATATATCTGTTTAACAAAGATAA
GATAACAGAAGAAGGTATGGATGGTTACTATGCTAGTGGTAACGGCGGCATTAT
T
>Seq32 [organism=organism=Aleutian mink disease virus] Aleutian mink disease virus isolate AMDV_IS_H32 non-structural protein 1 gene (NS1), partial cds
CTTTGATAAAGATGAAGATCCTAAGGATATTCAAAAATCCTTAGGTTGGTTTATT
AAAAAACTAAGTAGAGACATAGCAGTTGTTTATAGTAACCATCATTGTGACCTGC
AAGGTATACAAGATTCTGAAGGTAGATCTAATAACTTGAAAGTATGGATTGAAG
ATGGACCTACCAAACCTTACAAATACTTTAACAGACAAACCAAACAAGAATACA
ACAAACCAGTAAACATGCGTGACTATACATTAATATATCTGTTTAACAAAAATAA
GATAACAGAAGAAGGTATGGATGGTTACTATGCTAGTGGTAACGGCGGCATGAT
T
>Seq33 [organism=organism=Aleutian mink disease virus] Aleutian mink disease virus isolate AMDV_IS_H33 non-structural protein 1 gene (NS1), partial cds
CTTTGATAAAGATGAAGATCCTAAGGATATTCAAAAATCCTTAGGTTGGTTTATT
AAAAAACTAAGTAAAGACTTAGCAGTTGTTTATAGTAACCATCATTGTGACCTGC
AAGATATACAAGATCCTGAAAGTAGATCTAATAACTTGAAAATATGGATTGAAG
ATGGGCCTACAAAACCTTACAAATACTTTAACAGACAAACCAAACAAGAATACA
ACAAACCAGTGAACGTACGTGACTATACATTAATATATCTGTTTAACAAAGATAA
GATAACAGAAGAAGGTATGGATGGTTACTATGCTGCTGGTAACGGCGGCATTAT
T
